# COVID-19 Pandemic Impact on Surgical Treatment Methods for Early-Stage Cervical Cancer: A Population-Based Study in Romania

**DOI:** 10.3390/healthcare10040639

**Published:** 2022-03-28

**Authors:** Alin Popescu, Marius Craina, Stelian Pantea, Catalin Pirvu, Daniela Radu, Iosif Marincu, Felix Bratosin, Iulia Bogdan, Samer Hosin, Cosmin Citu, Elena Bernad, Radu Neamtu, Catalin Dumitru, Adelina Geanina Mocanu, Adrian Gluhovschi

**Affiliations:** 1 Department of Obstetrics and Gynecology, “Victor Babes” University of Medicine and Pharmacy, 300041 Timisoara, Romania; alinp22@yahoo.com (A.P.); mariuscraina@hotmail.com (M.C.); citu.ioan@umft.ro (C.C.); ebernad@yahoo.com (E.B.); radu.neamtu@umft.ro (R.N.); dumcatal@yahoo.com (C.D.); adelinamocanu2955@yahoo.com (A.G.M.); adigluhovschi@yahoo.com (A.G.); 2 Department of General Surgery, “Victor Babes” University of Medicine and Pharmacy, 300041 Timisoara, Romania; pirvu.catalin.alexandru@gmail.com (C.P.); daniela_radu@hotmail.com (D.R.); 3 Methodological and Infectious Diseases Research Center, Department of Infectious Diseases, “Victor Babes” University of Medicine and Pharmacy, 300041 Timisoara, Romania; imarincu@umft.ro (I.M.); felix.bratosin7@gmail.com (F.B.); iulia.georgianabogdan@gmail.com (I.B.); 4 Department of Orthopedics, “Victor Babes” University of Medicine and Pharmacy Timisoara, 300041 Timisoara, Romania; samerhosin@gmail.com

**Keywords:** SARS-CoV-2, COVID-19, cervical cancer, hysterectomy, disease-free survival

## Abstract

Being one of the most common malignancies in young women, cervical cancer is frequently successfully screened around the world. Early detection enables for an important number of curative options that allow for more than 90% of patients to survive more than three years without cancer relapse. Unfortunately, the COVID-19 pandemic put tremendous pressure on healthcare systems and access to cancer care, determining us to develop a study on the influence the pandemic had on surgical care of cervical cancer, and to assess changes in its management and outcomes. A retrospective study design allowed us to compare cervical cancer trends of the last 48 months of the pre-pandemic period with the first 24 months during the COVID-19 pandemic, using the database from the Timis County Emergency Clinical Hospital. New cases of cervical cancer presented to our clinic in more advanced stages (34.6% cases of FIGO stage III during the pandemic vs. 22.4% before the pandemic, *p*-value = 0.047). These patients faced significantly more changes in treatment plans, postponed surgeries, and postponed radio-chemotherapy treatment. From the full cohort of cervical cancer patients, 160 were early stages eligible for curative intervention who completed a three-year follow-up period. The disease-free survival and overall survival were not influenced by the surgical treatment of choice, or by the SARS-CoV-2 infection (log-rank *p*-value = 0.449, respectively log-rank *p*-value = 0.608). The individual risk factors identified for the three-year mortality risk were independent of the SARS-CoV-2 infection and treatment changes during the COVID-19 pandemic. We observed significantly fewer cases of cervical cancer diagnosed per year during the first 24 months of the COVID-19 pandemic, blaming the changes in healthcare system regulations that failed to offer the same conditions as before the pandemic. Even though we did not observe significant changes in disease-free survival of early-stage cervical cancers, we expect the excess of cases diagnosed in later stages to have lower survival rates, imposing the healthcare systems to consider different strategies for these patients while the pandemic is still ongoing.

## 1. Introduction

Cervical cancer is the second most common cancer in young women, being responsible for more than two hundred thousand deaths worldwide [1]. It starts as premalignant lesions confined to the cervix uteri that further develop to the parametrium, uterus, and vagina. There is an initial lymphatic spread to the obturator, external and internal iliac, continuing to involve the para-aortic lymph nodes, while distant metastasis occurs in later stages of the disease by hematogenous spread, most commonly to lungs, liver, and bones [2,3]. The Surveillance, Epidemiology, and End Results (SEER) staging of the American Cancer Society groups cervical cancer as localized, regional, and distant, while the International Federation of Gynecology and Obstetrics (FIGO) is the most developed staging system currently available, ranging from stage I to stage IVB [4]. FIGO defines the early-stage cervical cancer as IA1, IA2, and IB1, while the recommendations stand for radical surgery for stages IA2-IB1, associated with radiotherapy in selected cases based on lymph node metastasis, local parametrial invasion, tumor size, deep stromal invasion, and positive surgical margins [5].

Curative treatment is mostly achieved in the early stages that are recommended for surgical removal. The classic surgery performed in these cases is the Wertheim–Meigs procedure, which stands for radical hysterectomy with pelvic lymphadenectomy in an open surgery fashion, which was implemented more than 100 years ago and has been slightly modified over time with the advances of surgical and medical practice [6]. The development of surgical robots allowed multiple minimally invasive procedures to be performed, including the robotic-assisted hysterectomy that has the same purpose as the Wertheim–Meigs procedure [7].

Several researchers have undertaken investigations using COVID-19 to describe cancer patients. Cancer affects around 1–2.5 percent of COVID-19 patients, and their death rate varies from 11.4 to 28.6 percent [8,9], which is much higher than the overall population. Regrettably, several studies overlooked the consequences of various forms of cancer. Cervical cancer, being one of the most frequent cancers in women, has a lengthy treatment cycle that results in immunosuppression and an increased risk of infection; hence, patients with cervical cancer are at a greater risk of COVID-19 infection and should be monitored closely [10]. Moreover, during full and partial lockdown periods, all non-essential medical and surgical activity was halted to increase capacity for managing COVID-19 patients, in order to avoid overloading intensive care units and to provide care in the safest possible manner to patients with other pathologies, particularly cancer patients, who have a five-fold risk of developing a severe illness and a four-fold risk of death in the event of COVID-19 infection [11]. However, these patients’ access to screening and cancer care was detrimental to the COVID-19 management plans, many cases failing to be diagnosed in a timely manner or being diagnosed at all [12,13].

Therefore, encountering a population with more advanced cervical cancer stages imposes different medical and surgical approaches and can affect the morbidity and mortality of these patients. In light of the multitude of changes and disruptions in the healthcare systems that appeared during the COVID-19 pandemic, we developed a study to determine the influence the pandemic had on surgical care of cervical cancer patients, and to assess changes in the management of these patients that might affect their survival.

## 2. Materials and Methods

### 2.1. Study Design and Ethics

Our study followed a retrospective cohort design at the University Clinic of Obstetrics and Gynecology “Bega” and the General Surgery Department of the Timis County Emergency Clinical Hospital “Pius Brinzeu” from Timisoara, Romania, affiliated with the “Victor Babes” University of Medicine and Pharmacy. The research population and characteristics of interest were identified using the population-based administrative database at the same clinic. Our centralized database includes patient medical records kept under privacy protection and patient consent. The study was spread between a four-year pre-pandemic period and a two-year pandemic period, from 1 January 2016 to 1 January 2020, and from 1 January 2020 until 1 January 2022, respectively.

The Local Commission of Ethics for Scientific Research from the Timis County Emergency Clinical Hospital “Pius Brinzeu” from Timisoara, Romania operates under article 167 provisions of Law no. 95/2006, art. 28, chapter VIII of order 904/2006 and with EU GCP Directives 2005/28/EC, International Conference of Harmonisation of Technical Requirements for Registration of Pharmaceuticals for Human Use (ICH), and with the Declaration of Helsinki—Recommendations Guiding Medical Doctors in Biomedical Research Involving Human Subjects. The current study was approved on 10 December 2021, with approval number 18.

### 2.2. Study Variables and Statistical Analysis

The study variables were stratified by the COVID-19 pandemic period and included demographic data, comorbidities, complications after surgery, FIGO staging, chemotherapy, radiotherapy, and follow-up results. Complications after surgery were classified according to the Clavien–Dindo scale [14]. Data from patients diagnosed with cervical cancer during the first year of this study, who completed a follow-up period of at least three years after surgical or medical cancer therapy, were used to forecast a disease-free survival analysis based on the surgical treatment type. The follow-up of patients was performed according to the recent guidelines criteria [15]. Follow-up by phone or e-mail to patients or patients’ relatives was a solution to determine the status of those who failed to attend checkup appointments. The follow-up visits included general physical and pelvic examination with cytology and imaging studies. The cytology test and abdominal computed tomography were performed once a year, according to existing standards and guidelines [16,17]. We collected information from our database for a total of 392 patients who satisfied inclusion criteria by presenting to our clinic with a cervical cancer diagnosis during the six-year study period.

Statistical analysis was performed using the IBM SPSS software version 26.0 (IBM, Armonk, NY, USA). Categorical variables were represented as absolute and percentage values. Student’s t-test was used to analyze Gaussian data reported by mean and standard deviation, while the Wilcoxon–Mann–Whitney U-test was used for nonparametric variables. The χ^2^ and Fisher’s exact tests were used for statistical analysis of proportions. A Kaplan–Meyer plot was created to determine the probability of the three-year overall survival based on status of SARS-CoV-2 infection. A secondary Kaplan–Meyer curve was plotted to determine if the three-year disease-free survival of patients operated for early-stage cervical cancer differ between robot-surgery and open surgery methods, in order to assess whether choosing a cheaper option with a curative intention for cervical cancer during the COVID-19 pandemic can have a negative impact on the future outcome. The log-rank test analyzed the statistical significance of the plotted curves. Lastly, we implemented a multivariate regression analysis of the factors associated with overall survival. The significance threshold was set for α = 0.05.

## 3. Results

We observed an average of 57 patients per year in the pre-pandemic period and 26 patients per year during the COVID-19 pandemic. However, the baseline characteristics of these patients did not significantly differ as before and during the pandemic, having a normally distributed age and BMI, as presented in Table 1. There were statistically significantly fewer cases of cervical cancer diagnosed during the pandemic, and more cervical cancers found in more advanced stages. A total of 21.1% of cervical cancers presenting during the pandemic were FIGO stage I, compared with 39.7% before the pandemic. FIGO stage III cervical cancers accounted for 34.6% of the total number during the first 24 months of the pandemic, compared with 22.4% before the pandemic (*p*-value = 0.047). It was also observed that newly diagnosed patients faced significantly more changes in treatment plans (12.1% pre-pandemic vs. 23.1% during the pandemic, *p*-value = 0.030), postponed surgeries (9.4% pre-pandemic vs. 21.2% during the pandemic, *p*-value = 0.011), and radio-chemotherapy treatment changes (12.9% pre-pandemic vs. 28.8% during the pandemic, *p*-value = 0.002), as shown in Table 1.

**Figure 1:**
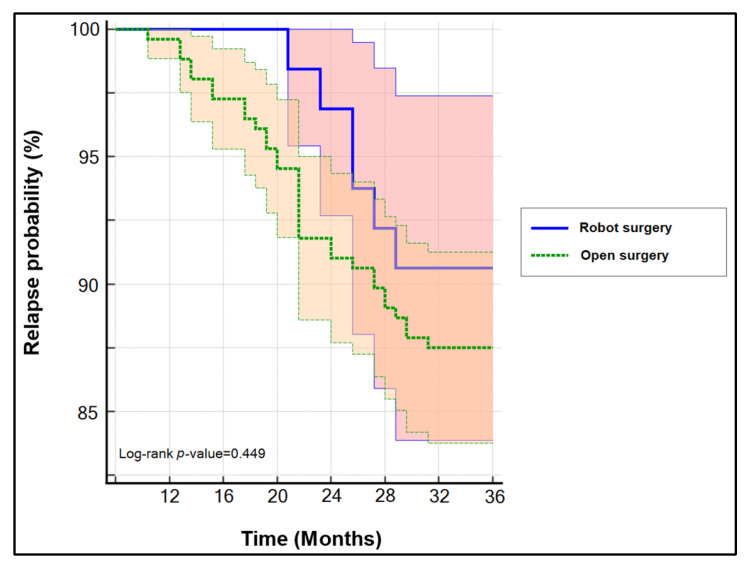
Kaplan–Meyer plot of the three-year disease-free survival in patients with early-stage cervical cancer based on the surgical treatment type.

**Figure 2:**
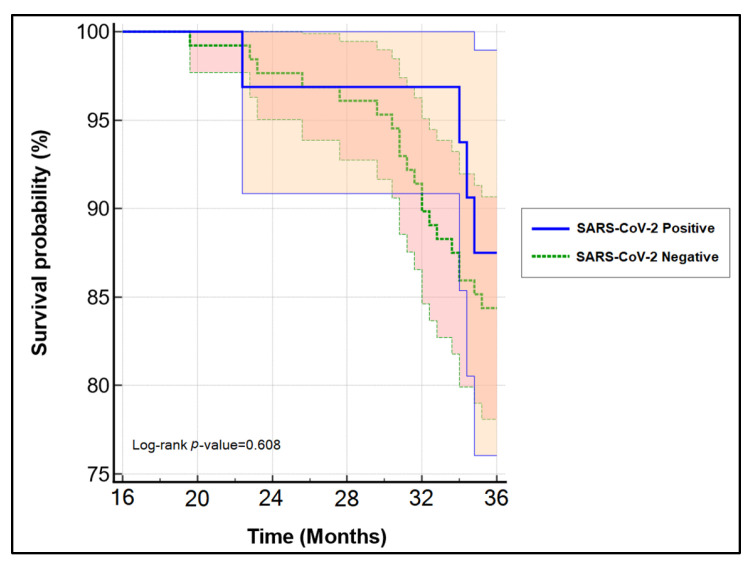
Kaplan–Meyer plot of the three-year survival probability in patients with newly-diagnosed cervical cancer based on SARS-CoV-2 infection status.

Among the 392 patients presenting with cervical cancer during the study period, we identified 160 early-stage cancers that underwent surgery with curative intentions and completed a follow-up period of three years. A total of 32 patients were infected with SARS-CoV-2 during the first 24 months of the COVID-19 pandemic. A stratification based on their SARS-CoV-2 status is presented in Table 2. Similar to the entire cohort, these patients did not show significant differences in their baseline characteristics and oncological findings. Twenty-four patients benefited from robotic-assisted hysterectomy, while the remaining had a classic Wertheim–Meigs hysterectomy with curative intentions. There were no significant differences in the three-year disease-free survival based on surgical treatment method shown in Figure 1 (log-rank *p*-value = 0.449), even though the Clavien–Dindo score was significantly lower for patients operated in minimally-invasive techniques (*p*-value = 0.031), and during the follow-up period, they had significantly more changes in treatment methods or delayed appointments caused by the COVID-19 restrictions. Moreover, the overall survival was not influenced in a significant manner by the SARS-CoV-2 status (log-rank *p*-value = 0.608) (Figure 2).

A risk factor analysis was performed to determine the independent causes for mortality at three years in patients newly diagnosed with cervical cancer during the study period. Table 3 describes our findings, identifying a large tumor size (≥2 cm), cancer relapse, high-grade histology type, and the number of lymph nodes involved (≥2) as significant independent risk factors for mortality. However, SARS-CoV-2 infection and a Clavien–Dindo score of 3 or higher did not pose as significant independent risk factors for mortality at three years (CI = 0.7–1.9, *p*-value = 0.246; respectively CI = 0.9–1.9, *p*-value = 0.085). The changes in medical or surgical plans, as well as postponed surgery and radio/chemotherapy that occurred during the COVID-19 pandemic, did not seem to be independent risk factors for negative outcomes.

## 4. Discussion

The current study provides a comprehensive analysis of the effects of the COVID-19 pandemic on medical and surgical management, disease-free survival, and overall survival in patients with early-stage cervical cancer, in the attempt of forecasting expectations for patients with this type of cancer during the pandemic and determining future implications. Another endpoint that we reached was to discover several risk factors for mortality in this cohort, such as the SARS-CoV-2 infection for patients with cervical cancer. Moreover, our study describes postoperative outcomes in comparison between two surgical approaches, the classic open surgery for radical hysterectomy with pelvic lymphadenectomy and the modern robotic surgery radical hysterectomy with pelvic lymphadenectomy. Several wider studies [18] have analyzed the same research question to compare the abdominal surgery versus a minimally-invasive surgical approach, although radical laparoscopic hysterectomy with pelvic lymphadenectomy replaced the robotic minimally-invasive method. In this nationwide study from the Netherlands, there was a higher mortality and cancer recurrence in the open abdominal surgery study arm, but there was no statistically significant difference in the disease-free survival and overall survival. However, an important difference was the lower rate of intraoperative and early complications from the minimally-invasive approach, including urogenital damage, infections, hemorrhage, or wound dehiscence, as previously described by multiple studies [19,20,21,22]. Therefore, it is important to determine what surgical method should be used in COVID-19 positive patients scheduled for a curative intervention because the options available in a “red zone” department can influence the long-term outcomes.

Although the three-year overall survival of FIGO stage I cervical cancer is reported to stand above 90% [23], our study reported lower overall survival (85%) that can be attributed to a higher incidence of higher-grade cancer (11%) and the effects of the COVID-19 pandemic on the Romanian national screening program [24,25], as well as treatment availability or SARS-CoV-2 infection in these patients. A recent comprehensive study [26] published the analysis of over 30 thousand women with cervical cancer, where the results indicated grade 3 tumors having a 4.48 hazard ratio (HR) as an independent association with a lower survival rate. Our study is in accordance with another research made on the management of early-stage cervical carcinoma through radical hysterectomy [27] that achieved similar results but included stages IA1 and IB2 in the study cohort.

The call for protective measures and strict restrictions imposed during partial and total lockdown led the gynecologists, surgeons, and hospitals to adapt their care strategies, resulting in significant disparities compared with the same period before the pandemic. In our study, we observed how cervical cancer patients had significantly more changes in treatment strategies during the pandemic and more medical and surgical interventions that were postponed, such as radiotherapy or chemotherapy. A change in the treatment plan was required mostly for SARS-CoV-2 positive patients who received cancer therapy in special conditions, under isolation in the COVID-19 “red zone” department. Other changes in treatment plans were the surgical method used because minimally invasive surgery was not available in the “red zone”. Similar findings were reported in two different European studies assessing disruptions in cancer care for gynecological cancers [28,29], wherein 30–40% of instances a change in chemotherapy approach was seen, as was a stop to laparoscopic surgery in 30% of cases and an increase in radiation treatment in 24% of cases. Moreover, even though surgery remained the primary therapeutic choice, it was postponed in 15% of instances for early-stage cervical malignancies. At the same time, the increase in missed cases and late-arriving patients with cancers will decrease the long-term survivability of these patients. Although multiple studies investigated the impact of screening failure for cervical cancer during the COVID-19 pandemic, fewer researches evaluated or estimated the survival of cervical cancer patients based on the treatment method or delayed treatment during the COVID-19 pandemic. A summary of these findings is presented in Table 4.

It is important to mention several advantages and limitations of the current study, as well as the implications involved. The main benefit that our study brings is consolidating the existing literature and setting a threshold for an accepted duration of treatment delay that will not compromise future outcomes. In terms of limitations, the sample size was rather small for a six-year study period, although the researchers were not in control of how many patients choose our clinic for specialized cancer treatment. In the same manner, a second limitation is the distribution of cases, where 160 of 392 eligible patients were early stages, operable with curative intentions. A third limitation would be the patient follow-up used to plot the survival curves because not all patients identified here were followed for three years. Lastly, the generalizability of this study can be considered for populations that share similar characteristics, cancer type, and cancer stage.

## 5. Conclusions

The pandemic thrust our world into an unprecedented healthcare crisis, necessitating rapid adaptation by the medical community, where the prompt reaction was necessary to enable the development of recommendations to aid clinicians. However, as our research demonstrates, this time period had a considerable influence on cervical cancer treatment tactics. Although the preferred surgical method between classic hysterectomy and robotic hysterectomy did not affect the disease-free survival of patients with early-stage cervical cancer, nor did the SARS-CoV-2 infection alter the overall survivability of these patients, changes in treatment were recorded in 31% of instances, and interventions were suspended in 25% of cases, depending on the healthcare provider’s availability and the patient’s SARS-CoV-2 status. Therefore, during and after the COVID-19 pandemic, practicing physicians should strictly avoid any delays longer than eight weeks in surgical and medical treatment for patients with cervical cancer that can detrimentally challenge the survival and disease-free survival. Complementing this work with further research on the impact of the COVID-19 pandemic on patient survival will provide further insight into the effectiveness of the lockdown rules and the number of diagnostic delays caused by the cancellation of several expert visits.

## Figures and Tables

**Table 1:** Characteristics of new patients with cervical cancer presenting at gynecology and general surgery clinics for investigations and treatment over a course of six years before and during the COVID-19 pandemic.

Variables *	Pre-Pandemic (*n* = 340)	During Pandemic (*n* = 52)	*p*-Value
Age, years (mean ± SD)	38.6 ± 8.1	37.4 ± 8.6	0.324
BMI, kg/m^2^ (mean ± SD)	23.3 ± 4.4	23.8 ± 5.2	0.457
**Tumor size, *n* (%)**			0.248
<2 cm	133 (39.2%)	16 (30.3%)	
≥2 cm	207 (60.8%)	36 (69.2%)	
**FIGO stage, *n* (%)**			0.047
I	135 (39.7%)	11 (21.1%)	
II	82 (24.1%)	13 (25.0%)	
III	76 (22.4%)	18 (34.6%)	
IV	47 (13.8%)	10 (19.2%)	
**Differentiation grade, *n* (%)**			0.942
Grade 1	191 (56.2%)	29 (55.7%)	
Grade 2	105 (30.9%)	17 (32.7%)	
Grade 3	44 (12.9%)	6 (11.6%)	
**Histology**			0.755
Squamous-cell	268 (78.8%)	40 (76.9%)	
Adenocarcinoma	72 (21.2%)	12 (23.1%)	
**Outcomes**			
Change in treatment plan	41 (12.1%)	12 (23.1%)	0.030
Postponed surgery	32 (9.4%)	11 (21.2%)	0.011
Postponed radio-chemotherapy	44 (12.9%)	15 (28.8%)	0.002
ICU hospitalization	23 (6.8%)	6 (11.5%)	0.220
Mortality	20 (5.9%)	4 (7.7%)	0.612

* Data reported as *n*(%) unless specified differently.

**Table 2:** Characteristics of patients with early-stage cervical cancer under follow-up based on their COVID-19 status.

Characteristics *	SARS-CoV-2 Negative (*n* = 128)	SARS-CoV-2 Positive (*n* = 32)	*p*-Value
Age, years	38.0 ± 9.4	39.2 ± 9.1	0.516
BMI, kg/m^2^	26.4 ± 3.5	27.8 ± 4.4	0.057
Overall survival	108 (84.4%)	28 (87.5%)	0.657
DFS **, months, (median[IQR])	34 [31–36]	33 [30–36]	0.531
Follow-up, months, (median[IQR])	34 [28–36]	34 [29–36]	0.948
**Surgical treatment type**			0.076
Robot surgery	16 (12.5%)	8 (25.0%)	
Open surgery	112 (87.5%)	24 (75.0%)	
**Tumor size**			0.871
<2 cm	50 (39.1%)	12 (37.5%)	
≥2 cm	78 (60.9%)	20 (62.5%)	
**Lymph node involvement**			0.141
0	94 (73.4%)	18 (56.3%)	
1	12 (9.4%)	6 (18.7%)	
>1	22 (17.2%)	8 (25.0%)	
**FIGO stage**			0.490
IA2	40 (31.3%)	8 (25.0%)	
IB1	88 (68.7%)	24 (75.0%)	
**Differentiation grade**			0.967
Grade 1	74 (57.8%)	18 (56.3%)	
Grade 2	40 (31.2%)	10 (31.3%)	
Grade 3	14 (11.0%)	4 (12.4%)	
**Relapse (*n* = 38)**			0.868
Local	16 (12.4%)	3 (9.4%)	
Regional	8 (6.3%)	2 (6.3%)	
Distant	8 (6.3%)	1 (3.2%)	
Total	32 (25.0%)	6 (18.8%)	
**Histology**			0.223
Squamous-cell	111 (86.7%)	25 (78.1%)	
Adenocarcinoma	17 (13.3%)	7 (21.9%)	
**Adjuvant treatment**			0.762
Radiotherapy-only	20 (15.6%)	8 (25.0%)	
Chemotherapy-only	5 (3.9%)	3 (9.4%)	
Radio-chemotherapy	6 (4.7%)	4 (12.4%)	
**Clavien-Dindo scale**	**Classic surgery**	**Robotic surgery**	0.031
No complications	45 (35.2%)	20 (62.5%)	
Score 1	52 (40.6%)	8 (25.0%)	
Score 2	24 (18.8%)	2 (6.25%)	
Score 3	7 (5.4%)	2 (6.25%)	

* Data reported as *n*(%) unless specified differently; ** DFS—Disease-Free Survival.

**Table 3:** Multivariate regression analysis for the three-years mortality in patients with early-stage cervical cancer.

Factor	Odds Ratio	Confidence Interval	*p*-Value
Tumor Size (≥2 cm)	1.8	1.4–2.5	0.022
Relapse	4.2	3.1–5.8	<0.001
High Grade	5.1	3.3–7.2	<0.001
SARS-CoV-2 infection	1.3	0.7–1.9	0.246
Lymph Nodes (≥2)	2.9	1.6–3.6	0.003
Clavien–Dindo (≥3)	1.5	0.9–1.9	0.085
Change in treatment plan	1.3	0.9–1.6	0.104
Postponed surgery *	1.1	0.8–1.3	0.417
Postponed radio-chemotherapy *	1.3	0.8–1.7	0.115

* Between 1 and 8 weeks.

**Table 4:** Existing studies describing the COVID-19 pandemic impact on cervical cancer survival.

First Author (Year)	Conclusions
Gupta et al. (2021) [30]	A 2.52% to 3.80% increase in cervical cancer-related deaths with treatment delays ranging from 9 weeks to 6 months.
Kregting et al. (2021) [31]	An increase of 2.0, 0.3, and 2.5 cancer deaths per 100,000 individuals in 10 years.
Matsuo et al. (2021) [32]	Wait-time of 6.1–9.8 weeks for cervical cancer treatment was not associated with increased risk of all-cause mortality compared to a wait-time of 6 weeks.
Matsuo et al. (2021) [33]	In women with early-stage cervical cancer, an 8-week delay for hysterectomy may not be related with short-term disease recurrence and shorter DFS.
Davies et al. (2022) [34]	Over the next 3 years, there is anticipated considerable rise in newly-diagnosed cervical cancer cases. Increased surgical capacity might alleviate this burden with no significant morbidity or mortality increase.
Matsuo et al. (2021) [35]	Postponing hysterectomy for 6–8 weeks is appropriate for women with early-stage cervical cancer in centers or areas with a high prevalence of COVID-19 illness and has no detrimental effect on survival.

## Data Availability

The data presented in this study are available on request from the corresponding author.
